# Teachers’ Lived Experiences of Workplace Violence and Harassment Committed by Learners from Selected High Schools in Limpopo Province, South Africa

**DOI:** 10.3390/healthcare11182602

**Published:** 2023-09-21

**Authors:** Madie Collen Mangena, Sogo France Matlala

**Affiliations:** Department of Public Health, University of Limpopo, Sovenga, Polokwane 0727, South Africa; mmangena3@gmail.com

**Keywords:** bullying, enabling environment, health-promoting school, occupational health and safety, safe school, teaching and learning, workplace violence, employee assistance program

## Abstract

Despite several studies on learner-to-teacher workplace violence and harassment, the problem persists in some South African schools. Learner-to-teacher violence and harassment is a form of workplace violence and harassment, as schools are workplaces for teachers. Learner-to-teacher violence and harassment is therefore an important occupational health and safety issue for teachers. Employers are obliged to provide a safe working environment for teachers to enable quality teaching and learning in schools. The purpose of this interpretative phenomenological study was to explore and describe the lived experiences of high school teachers who have been targets of workplace violence and harassment perpetrated by learners at selected schools in Limpopo Province of South Africa. Many teachers were willing to share their lived experiences but, due to data saturation, only eleven participated after being selected through purposive sampling from seven high schools under a chosen sub-district. The research ethics of voluntary participation, informed consent, ethical clearance, and gatekeeper permission were observed. Data were collected through semi-structured interviews using an interview guide. The interviews were audio-taped, and field notes were also taken. Voice recordings were transcribed verbatim and analysed using interpretative phenomenological analysis into themes and sub-themes. The findings were confirmed by an independent coder to achieve trustworthiness. Teachers experienced physical workplace violence and harassment, verbal workplace violence and harassment, and nonverbal workplace violence and harassment from learners. Learner-to-teacher workplace violence and harassment affects teachers emotionally, and in turn, affects the quality of teaching and learning in schools. Some teachers propose the involvement of a community policing forum, the strengthening of schools’ governing bodies, and reducing overcrowding in classrooms as possible solutions to deal with learner-to-teacher workplace violence and harassment.

## 1. Introduction

The International Labour Organization (ILO) defines workplace violence and harassment (WVH) as various forms of violent actions or threats, whether a single incidence or a repetitive one, that aim at, result in, or are likely to result in physical, psychological, sexual, or economic harm to a worker. This refers to workers in the formal and informal economy, in the public and private sectors, and any worker irrespective of his or her contractual status [1]. WVH poses threat to the health and safety of workers and can result in physical and mental harm; as such, the ILO calls for all its member countries to create workplaces that promote and protect the health and psychophysical wellbeing of workers [2]. The government of South Africa enacted the Occupational Health and Safety Act No. 85 of 1993 to promote and protect the health and psychophysical wellbeing of workers. The Act requires workplaces to have health and safety policies [3].

WVH occurring in schools can be in the form of learner-to-teacher violence and harassment or teacher-to-teacher violence and harassment. In teacher-to-teacher violence and harassment, peers commit violence and harassment to each other horizontally, while in learner-to-teacher violence and harassment, the process is vertical as teachers and learners are not peers. In all these forms of violence and harassment, the action is perpetrated by the seemingly powerful people to the seemingly powerless ones who are unable to defend themselves [4,5]. Our study focuses on learner-to-teacher violence and harassment which involves the harassment of an adult by a child. In this case, the child is the learner while the adult is the teacher. In South Africa, the age of learners in secondary schools ranges between 15 and 18 years. WVH that targets teachers and is committed by learners refers to various forms of aggressive behaviours that may include insults, inappropriate comments, disrespect, shouting and yelling at, bullying and verbal threats, harassment through the internet, damage to or theft of personal property, physical assault, and disregard for teachers’ instruction [6]. Furthermore, WVH can manifest as malicious damage to the victim’s property, pestering, intimidations, verbal abuse, and physical attacks [7].

Learner-to-teacher workplace violence and harassment (LTWVH) is a challenge in South Africa and is reported in the media regularly [8,9,10]. Media reports further show that LTWVH is a challenge in other countries as well [11,12,13,14,15]. Research shows that LTWVH is a global problem. Teachers from the United States of America, Canada, the United Kingdom, and Israel shared their experiences of LTWVH [16,17,18,19,20]. Studies conducted in South Africa show that schools, both primary and secondary, are becoming unsafe workplaces for teachers as they experience various forms of LTWVH [21,22,23]. All these types of WVH are harmful to the health and psychophysical wellbeing of teachers.

The problem of LTWVH continues despite several measures and suggestions to manage it. The aim of this study is to explore and describe experiences of secondary school teachers who were victims of LTWVH to further explore it and contribute to its understanding. The structure of this paper is guided by the Consolidated Criteria for Reporting Qualitative studies (COREQ) and the Standards for Reporting Qualitative Research (SRQR) [24,25]. These reporting guidelines are relevant for our study as the COREQ is suitable to use in studies that use focus group discussions and interviews; our study used interviews, while the SRQR is suitable for all types of qualitative studies [26].

### 1.1. Theoretical Framework

Both the COREQ and the SRQR recommend that researchers describe the theory or theories that guide their studies so that readers can understand how the research questions and the objectives were explored [24,25]. This study is guided by the Health Promoting Schools Framework as well as the National School Safety Framework [27,28]. The two frameworks share similarities regarding the management of WVH in schools. A health-promoting school is a school which is continuously strengthening its capacity to promote and protect the health and psychophysical wellbeing of both teachers and learners [29]. Prinsloo defines a safe school as a school that is free from acts of violence and harassment, while also having plans to deal with them should they occur [30]. A safe school is also a health-promoting school; as such, the Health Promoting Schools Framework and the National School Safety Framework complement each other in creating a healthy setting for teachers to work and for learners to learn. Elements of the two frameworks relevant to management of LTWVH are briefly described below.

#### 1.1.1. The Health Promoting Schools Framework

The Health Promoting Schools Framework has four pillars, which are the school environment; curriculum and learning; partnerships; and policy and planning [27]. The four pillars are described below in as far as they apply to workplace violence and harassment at school.

- School environment

The school environment refers to the social environment and the psychological environment. The social environment of a school is about relationships between teachers and learners as well as with management. The psychological environment is about schools’ code of conduct, and the support services for both learners and teachers. Promoting and protecting the health and psychophysical wellbeing of both teachers and learners requires teachers and learners to work together while acknowledging the power relations between them.

- Curriculum and learning

Curriculum and learning refers to addressing violence and harassment through the curriculum via the inclusion of life skills and education on violence and harassment in school subjects. Violence and harassment in the school curriculum can include a school subject such as Life Orientation that teaches learners to identify forms of violence and harassment, and be empowered on how to respond when they are harassed. Empowered learners will not perpetrate violence and harassment towards their teachers or towards other learners at their schools.

- Partnerships

Partnerships refer to schools establishing relationships with relevant stakeholders from the community to address workplace violence and harassment in their schools. Relevant stakeholders include the community policing forum (CPF), social workers, and nongovernmental organizations (NGOs).

- Policy and planning

Policy and planning refer to the existence of school policies to address violence and harassment. This refers to actions to prevent violence and harassment as well as a readiness to act in case they occur.

#### 1.1.2. The National School Safety Framework

The National School Safety Framework aims to promote and protect the health and psychophysical wellbeing of both teachers and learners by addressing incidences of LTWVH. This framework indicates that the optimal health and psychophysical wellbeing of teachers and learners is a requirement for quality teaching and learning to take place. Just like the Health Promoting Schools Framework, the National School Safety Framework also has four pillars, which are a willingness to prevent and manage safety-related problems; an awareness of the safety climate of the school; a readiness to act; and building a caring school [28].

- Willingness to prevent and manage safety-related problems

Schools should have updated codes of conduct, safety policies, and procedures for addressing violence and harassment when they arise.

- An awareness of the safety climate of the school

Every school should be aware of its level of safety as well as its resources for making the school safe. There should be regular monitoring, evaluation, and control activities to ensure that violence and harassment are prevented and managed properly if detected.

- Readiness to take action

A school should be ready to respond to early warning signs of workplace violence and harassment. To be able to detect warning signs early, the school should make safety part of its cultural norm. Policies and procedures to manage violence and harassment should be available so that they are implemented in response to early warning signs.

- Building a caring school

A caring school is one that promotes and protects the health and psychophysical wellbeing of teachers and learners so that the two listen to and protect each other [31]. Building a caring school calls for the collective effort of all stakeholders such as the CPF, social workers, and NGOs.

## 2. Materials and Methods

The SRQR requires qualitative researchers to specify the qualitative approach used, while the COREQ calls for researchers to state the methodological orientation guiding their studies. This study is an example of interpretative qualitative research, and because it is about lived experiences, it is an interpretative phenomenological analysis (IPA) or interpretative phenomenology [32,33]. Interpretative phenomenology is informed by philosophers such as Heidegger, Gadamer, and Ricoeur. It strives to openly communicate a deeper understanding of the hidden meanings of everyday life experiences, unlike descriptive phenomenology, guided by the work of Edmund Hussel, with its focus on the life world, which just describes obvious meaning [33,34,35].

We chose to carry out an IPA for our study on learner-to-teacher workplace violence and harassment, as it would enable us to have a deeper understanding of the meaning of workplace learner-to-teacher violence and harassment as experienced by teachers in their lives as workers as well as in their social lives. As Smith et al. indicate, the importance of interpretative phenomenological analysis is its ability to reveal deeper meanings that certain experiences or events have for participants [32]. The purpose of interpretative phenomenological analysis is to search for deeper meanings that participants attach to events in their personal and social lives. Ahmad and Sheehan also used an interpretative phenomenological analysis in their research on workplace violence and harassment in Australia amongst a range of workers [4].

In interpretative phenomenological research, theory can be used in many ways. Firstly, a theory can be used to guide how a phenomenon is explored by shaping the type of questions that the researcher asks the participants. Secondly, it can be used as an analytical framework to extract themes deductively or to refine themes that were extracted inductively. Finally, researchers can use a theory to retrospectively make sense of data which were collected without following a theory [33]. Our study used the Health Promoting Schools Framework and the National School Safety Framework to refine themes that were extracted inductively to make sense of data that were collected without following a particular theory.

### 2.1. Ethical Considerations

This study obtained ethical clearance from the University of Limpopo (TREC/302/2020:PG), while the Limpopo Department of Basic Education gave gatekeeper permission. With the ethical clearance and the gatekeeper permission, the first author (MCM) visited the sub-district manager to request permission to go to schools to plan for data collection. The researchers respected the anonymity and confidentiality of teachers and the schools, as names are not mentioned. During the interviews, the researcher (MCM) requested permission from the participants to use a voice recorder and asked the participants not to mention the names of people and schools as they shared their experiences. After transcribing the data, both researchers (MCM and SFM) looked for information that could identify the participants or their schools and removed it. Research suggests that asking victims to describe their lived experiences of workplace violence and harassment might arouse negative emotions, as workplace violence and harassment is a sensitive issue [4]. We arranged with local social workers to be ready to offer counselling in case some teachers became distressed when relating their lived experiences of workplace violence and harassment committed by learners, but none showed signs of distress necessitating a referral to counselling both during and after the interviews.

### 2.2. Trustworthiness

We ensured trustworthiness by implementing credibility, conformability, dependability, and transferability principles [36]. We reported participants’ demographics, and themes which were confirmed by an independent coder to demonstrate that the study findings represent the views of the participants, and that the meaning we arrived at is correct. Our description of the method that was followed is detailed to enable other researchers to review the way we conducted the study.

### 2.3. Population and Sampling

As the COREQ and the SRQR recommend, this section describes the method and justification for selecting the participants. The population was secondary school teachers who experienced workplace violence and harassment committed by learners while working at seven secondary schools under the chosen sub-district in Limpopo Province of South Africa. The first author (MCM) became aware of this problem as some schools had asked for his professional intervention to address violence and harassment in their schools. The sample was made up of teachers who volunteered to participate in response to an invitation from the researchers. The first author (MCM) gave information about the study to teachers at all seven secondary schools and invited volunteers who experienced violence and harassment to share their experiences. Many teachers volunteered to share their experiences but only 11 were interviewed due to the saturation of data. During an interview, the saturation of data refers to a point where a researcher begins to hear what other participants have already shared. This signals to a researcher that further interviews should not be conducted as no new information will be discovered [35]. Eleven is a reasonable sample size for interpretative qualitative research, as Smith et al. recommend a small sample of between 10 and 20 participants to enable researchers to have meaningful interactions with each participant [32]. We used purposive sampling to select a heterogeneous sample to obtain experiences from male and female teachers of various age groups and teaching experience levels from all seven secondary schools in the chosen sub-district. In purposive sampling, the researcher sets a sampling criterion then selects participants who fulfil those criteria [35]. Teachers with more than two years of teaching experience and who had at least two meaningful experiences of being a target of violence and harassment by learners were invited to participate in the study. We regarded two years of teaching experience as sufficient for one to have experienced workplace violence and harassment perpetrated by learners and to have rich information to share. Table 1 shows the demographic data we planned to collect.

### 2.4. Data Collection

Collecting data for interpretative phenomenological research is a dynamic process, during which researchers are actively participating in an interpretative activity by trying to understand an experience from the point of view of the person who experienced it. It is a two-phase process where an outsider (the researcher) is trying to make sense of an insider (the participant) who is trying to make sense of his or her own experience [37]. To achieve this, researchers must acknowledge their assumptions and biases about the phenomenon being researched and then use them during data collection and analysis instead of bracketing or putting them away [32,34,35]. The COREQ and the SRQR recommend that researchers be open about their qualifications, experiences, relationships with participants, and their personal interests in the phenomenon being researched, as these may influence the way data are collected and analysed. In our study, the first author (MCM) is a male social worker who is regularly invited to schools to address psychosocial issues, which included violence and harassment. He received training and supervision to conduct qualitative research from the second author (SFM). The second author (SFM), also male, is experienced in qualitative health research, and has trained and supervised emerging qualitative researchers in the field of public health. The two researchers have an interest in school violence and harassment as a component of school health services. The provision of school health services is an important area in public health, aiming at making all schools to be health-promoting schools, as envisioned by the World Health Organization [38].

Data were collected through semi-structured interviews, using an interview guide, during which a central question “*What are your experiences of being a target of violence and harassment by learners at school*?” was asked to all participants. Some probing or follow-up questions were asked to participants depending on the depth of their answers to the central question. Table 1 is an interview guide showing the demographic data to be collected, as well as the central and probing questions. We did not conduct a pilot test as semi-structured interviews allow the researcher to rephrase and modify the questions during the interviews, guided by their understanding of the questions and the responses of each participant [32]. The duration of the interviews was between 40 and 60 min at places that were convenient to the participants, as individual appointments were made with each teacher who agreed to participate. All interviews were audio recorded and field notes were taken immediately after each interview to record nonverbal observations. One interview was conducted per day to permit a preliminary analysis before conducting the next one. This allowed us to identify issues to probe in the next interview; as such, there was no need to conduct repeat interviews. We conducted two rounds of interviews to ensure that we reach all seven high schools and heard experiences of both males and females, as well as teachers of various age groups and teaching experiences. During the first round, we interviewed one teacher from each school, and in the second round, we tried to interview teachers of different genders and age groups from those we had interviewed during the first round. The interviews were conducted at various locations, chosen by each participant. Due to the saturation of data and the limited availability of the various types of participants, we could not achieve the maximum variations in the sampling, as we had intended.

### 2.5. Data Analysis

In qualitative research, a preliminary data analysis begins during data collection so that researchers can detect the saturation of data and stop further interviews [39]. The first author (MCM) transcribed the audio recordings verbatim and added field notes to each transcript to prepare for the analysis process. We then looked for identifying particulars in the transcripts and removed them to ensure that the data could not be linked to the participants or their schools. Data analysis in IPA follows steps, although it is not a linear process. It involves moving forward and backward between the steps. We followed the steps described by Ahmad and Sheehan, which involve a familiarization with the data, an immersion in the data, categorization, pattern recognition, interpretation, and explanation [4]. We analysed each transcript independently and then discussed our findings to arrive at the themes. We also had a researcher, who was experienced in qualitative research, analyse the transcripts independently and hold a meeting to agree on the final themes to improve the credibility of the findings through triangulation.

## 3. Results

The demographic data (Table 2) shows that six female and five male teachers of different age groups and years of experience as teachers, working at seven different schools, participated. These data show that LTWVH occurred amongst various types of teachers and that these shared experiences represent a diverse group. This suggests that LTWVH is not a problem of a particular type of teacher, but it can happen to any teacher, thus making it difficult to give a description of teachers who are likely to be targets. Three themes with five subthemes emerged from the data analysis and are summarized in Table 2.

### 3.1. Theme 1: Forms of Learner-to-Teacher Workplace Violence and Harassment

This theme describes the different forms of LTWVH that teachers reported to have observed or experienced personally. Some observed or experienced physical LTWVH, while others experienced it as verbal or nonverbal, as described in the three sub-themes below.

- Sub-theme 1.1: Encounters of learner-to-teacher physical workplace violence and harassment

Some teachers reported being touched inappropriately by learners, while others were grabbed by learners in the presence of other learners in a classroom. A teacher who observed LTWVH said, ‘*The learner grabbed the teacher with his tie and pulled him … he wanted to suffocate him*’. A female teacher who experienced LTWVH personally said, ‘*eish, I remember one day a male learner touched my breast in class… and when I try to find out what was going on with him, the learner told me that I am nice and went he walked out of the classroom……*’

- Sub-theme 1.2: Encounters of learner-to-teacher nonverbal workplace violence and harassment

A teacher experienced nonverbal LTWVH by being shown a middle finger, while another experienced an intimidating look from a learner. All these happened in the presence of other learners. One teacher said, ‘*the learners laughed … later I asked what they were laughing for. They told me this learner showed a middle finger when I was facing the chalkboard*’. Another said, ‘*Those learners treated me badly outside the classroom … in the classroom they gave me a nasty look, intimidated me and they wanted to show other learners how unimportant I was*’.

- Sub-theme 1.3: Encounters of learner-to-teacher verbal workplace violence and harassment

A teacher reported that a learner yelled vulgar words while another reported about a learner who was known to insult teachers at a school. A teacher who experienced LTWVH personally said, ‘…the learner started shouting at me “fuck you! Go to hell! What can you do to me?’ Another added, ‘*I remember there was this learner who insulted teachers … every teacher who went to that classroom complained about him*’.

### 3.2. Theme 2: Impact of Learner-to-Teacher Workplace Violence and Harassment on Teachers

This theme describes the negative impact that LTWVH has on the health and psychophysical wellbeing of teachers. Teachers whose health and psychophysical wellbeing are impacted negatively perform their duties poorly. This is discussed in the following two sub-themes.

- Sub-theme 2.1: Impact on the health and psychological wellbeing of the teachers

A teacher reported on his emotional pain, anxiety, reluctance to go to a particular class, and the caution he adopted when interacting with learners. Another felt demeaned and humiliated by the actions of some learners. A teacher said, ‘*It was painful, very painful, each time I thought of going to that class I felt discouraged, and when I was busy teaching, I would be cautious of the words I say … I was anxious…*’ Another added, ‘*The learner insulted me in front of other learners…… it was a bit humiliating, a little bit embarrassing……*’.

- Sub-theme 2.2: Impact of learner-to-teacher workplace violence and harassment on performance of duties by the teachers

A teacher shared how thinking about going to teach in a class where there is an annoying learner dampens her spirit, while another wished he could be transferred to a different class to avoid teaching in a class where there was a rude learner. A teacher said, ‘*I felt as if I could change that class and give it to another teacher, but other learners should not suffer because of one learner. But my interaction is not the same as before. I am no longer laughing and joking, I just teach and then leave the classroom*’. Another said, ‘*Sometimes when this learner was in class, he would be troublesome. As a teacher I went in class being energetic, I wanted to teach this and that, but you know what… he would just spoiled my lesson*’.

### 3.3. Theme 3: Propositions to Resolve Learner-to-Teacher Workplace Violence and Harassment

Some teachers shared their experiences with LTWVH and made suggestions for stakeholders to resolve this challenge. One suggested the involvement of law enforcement to deal with unruly learners, while another felt that reducing overcrowding in classrooms could solve the problem. A teacher said, ‘*…call the police forum to come and assist us with those learners who are troublesome*’. Another added, ‘*I think the department could try to alleviate the problem of overcrowding, like what has been done with classes during COVID-19… we were able to get cooperation from learners’*.

## 4. Discussion

The aim of this study was to explore and describe the lived experiences of teachers with LTWVH. Furthermore, teachers shared their views on managing LTWVH. Teachers related their experiences of being targets of LTWVH, which presented as physical violence and harassment, nonverbal violence and harassment, and verbal violence and harassment. Physical violence and harassment occurred when a learner grabbed the teacher with his tie and pulled him as if wanting to suffocate him. In other studies, learners committed violence and harassment towards teachers by threatening them with violence and stealing or damaging their belongings, throwing objects at them and pinching them, as well as shoving them, pulling their hair and even touching them inappropriately [6,7,16,17,18,19,20,21,22,23]. Workplace violence and harassment occurs where there are power imbalances and, supposedly, teachers have more power compared to learners. In learner-to-teacher violence and harassment, the power imbalances are reversed, as the learner becomes more powerful than the teacher due to the expectation of the professional behaviour of the teacher as demanded in the teaching profession [40]. It is a case of a child being more powerful than an adult. It can therefore be assumed that learners who commit violence and harassment against teachers see themselves as having more power over the teachers they are targeting. A learner can commit violence and harassment against a teacher physically if he is aware that the teacher cannot retaliate due to legal and professional expectations of teacher conduct. The Code of Professional Ethics for Teachers expects every teacher to exercise power with restraint to avoid humiliation and physical or psychological harm to the learners [41]. The expected professional behaviour somehow makes teachers defenceless to workplace violence and harassment, as learners are aware that teachers will not hit back. Menesini and Salmivalli [42] confirm that a perpetrator of violence and harassment may achieve power by being aware of someone’s helplessness and then use that awareness to harm him or her.

Nonverbal WVH refer the use of body language or gestures to send hurtful messages to someone [5]. In our study, nonverbal LTWVH occurred when a learner showed a middle finger to their teacher, causing other learners in the class to laugh. The teacher did not see the middle finger signal but heard other learners giggling, who then informed the teacher that the other learner showed a middle finger signal. The learner achieved power over the teacher as the other learners in the class saw the gesture and laughed at the teacher. In other studies, nonverbal LTWVH was shown through rolling of the eyes, facial expression, and other hand signals that show some form of disgust, as well as making offensive gestures and threatening facial expressions or eye contact [22,23]. Showing someone a middle finger is generally accepted as an attacking sign to show disrespect or disobedience [43]. The teacher in our study felt attacked and disrespected by the disrespectful learner, and that lack of respect could also be displayed by other learners in the class if they realized that the teacher appeared powerless to punish their disrespectful classmate. It is for this reason that the National School Safety Framework [28] was drawn to create a safe and supportive learning environment in schools by addressing incidences of violence and harassment between learners and teachers. To achieve this, schools should have the capacity to identify violence and harassment as early as possible and take action to stop it.

In this study, verbal LTWVH was experienced when a learner insulted teachers. In other studies, teachers experienced violence and harassment by being teased, impersonated, being called funny names, and having learners make disgusting and hurtful comments about their character or personal appearance [21,22,23]. In our study, a teacher was a target of LTWVH by a learner who was known at school to be insulting to teachers. This implies that this learner was perceived by other learners as having more power than the teachers.

Teachers who experienced LTWVH related its negative impact on their health and psychophysical wellbeing, and ultimately on their ability to perform their teaching duties. It is not amazing for teachers to be distressed when learners commit violence and harassment against them, as LTWVH is an example of a child harassing an adult. Studies reveal that workplace violence and harassment affects the mental health of workers negatively and, as such, leads to low productivity [4,16]. In schools, WVH causes serious and sometimes long term psychosocial and professional harm to teachers. Professional harm is a result of, among others, the poor performance of teaching responsibilities, as teaching is a profession whose aim is to facilitate teaching and learning in the schools [21]. WVH has a negative impact on the health and psychophysical wellbeing of teachers, and results in the poor performance of teaching responsibilities. Some teachers who experienced LTWVH felt humiliated, disempowered, and did not want to go to work anymore [21,22,23]. Due to the negative impact of WVH on the health and psychophysical wellbeing of teachers, some have even left the profession as they could not continue working in a profession where their confidence and authority were undermined. LTWVH is detrimental to the social lives of teachers who, because of mental and professional harm, may feel disgraced and lose their ability to support themselves and their families financially [23].

Teachers who experienced LTWVH shared some propositions to address it. Some proposed an involvement of the CPF, social workers, and psychologists, while others proposed that reducing overcrowding in classrooms to a manageable number of learners might be helpful. These propositions are aligned to the four pillars of the National School Safety Framework, which are a willingness to prevent and manage safety-related problems; an awareness of the safety climate of the school; a readiness to act; and building a caring school [28]. The four pillars of the National School Safety Framework share similarities with those of the Health Promoting Schools Framework. These four pillars are the school environment; curriculum and learning; partnerships; and policy and planning [27]. These propositions show that teachers are eager to promote and protect the health and psychophysical wellbeing of teachers; are aware of the safety environment at their schools; and are ready to act by forming partnerships with the CPF to deal with LTWVH, as other activities for the promotion and protection of health and psychophysical wellbeing require a joint determination from all stakeholders. Building a caring school calls for a curriculum that will empower learners to identify and refrain from committing LTWVH. The Department of Basic Education, as the employer of teachers in secondary schools, should ensure that schools promote and protect the health and psychophysical wellbeing of teachers so that they can teach and enjoy going to school, as required by the Occupational Health and Safety Act 85 of 1993 [3]. The department should also establish an Employee Assistance Program (EAP) to support teachers who are traumatized and reluctant to go to some classes where they have been targets of violence and harassment. Furthermore, school governing bodies (SGB) should be empowered to prevent and manage LTWVH.

## 5. Conclusions

This study discussed LTWVH using an interpretative phenomenological design. Teachers, who are adults, shared their experiences of being targets of verbal, nonverbal, and physical LTWVH by learners, who are children that teachers are meant to guide and support as they become adults. The experiences devastated teachers in many respects, both socially and professionally. This study contributes to the knowledge on the existence of LTWVH in secondary schools and its negative impact on the health and psychophysical wellbeing of teachers, as well as on teaching and learning. It has demonstrated the use of the Health Promoting Schools Framework and the National School Safety Framework to extract meaning from data which were collected without following a theory. There is, therefore, a need to prevent and manage LTWVH to promote and protect the health and psychophysical wellbeing of teachers, as well as that of learners who witness their teachers being targets of LTWVH.

## 6. Limitations

Data collection took place during the COVID-19 pandemic, where direct social contact between the researchers and the participants was partial. This affected the duration and the way in which the interviews were conducted, as well as the willingness of some teachers to participate. Conducting virtual interviews was not possible at that time due to a lack of capacity.

## Figures and Tables

**Table 1:** Interview guide showing the type of data to be collected.

**Gender**	Female:				Male:		
**Age**	31 to 40:		41 to 50:			Older than 50:	
** School **	A:	B:	C:	D:	E:	F:	G:
** Teaching Experience **		Less than 10:		11 to 20:		21 to 30:	31 to 40:
**Central Question:** What are your experiences of being a target of violence and harassment by learners at school?							
**Follow-Up Questions:** What is your understanding of violence and harassment? Share with me how learners committed violence and harassment against you. In what way has violence and harassment affected your health and wellbeing? What do you find is stressing and impeding you from doing your work as a teacher? What do you suggest can be solutions to this problem of learner-to-teacher violence and harassment?							

**Table 2:** Demographic profile of participants and themes with sub-themes.

**Gender**	Female: 6				Male: 5		
**Age**	31 to 40: 1		41 to 50: 7			Older than 50: 3	
**School**	A: 2	B: 1	C: 2	D: 1	E: 1	F: 2	G: 2
**Teaching Experience**		Less than 10: 1		11 to 20: 3	21 to 30: 5		31 to 40: 2
**Themes**					**Sub-Themes**		
1. Forms of learner-to-teacher workplace violence and harassment					1.1 Encounters of learner-to-teacher physical workplace violence and harassment 1.2 Encounters of learner-to-teacher nonverbal workplace violence and harassment 1.3 Encounters of learner-to-teacher verbal workplace violence and harassment		
2. Impact of learner-to-teacher workplace violence and harassment					2.1 Impact of learner-to-teacher workplace violence and harassment on the health and psychological wellbeing of the teachers 2.2 Impact of learner-to-teacher workplace violence and harassment on performance of duties by the teachers		
3. Propositions to resolve learner-to-teacher workplace violence and harassment							

## Data Availability

The data presented in this study are available on request from the corresponding author.
